# PLATE USING CANCELLOUS GRAFT VERSUS SCREW USING CANCELLOUS CORTICAL GRAFT IN THE TREATMENT OF SCAPHOID PSEUDARTHROSIS

**DOI:** 10.1590/1413-785220253304e290649

**Published:** 2025-09-08

**Authors:** Erick Yoshio Wataya, Antonio Isidoro Sousa, Thales Augusto Tomé, Joao Carlos Nakamoto, Marcelo Rosa de Rezende, Rames Mattar

**Affiliations:** 1 Universidade de Sao Paulo, Faculdade de Medicina, Instituto de Ortopedia e Traumatologia (IOT HCFMUSP), Grupo de Cirurgia da Mao e Microcirurgia Reconstrutiva, Sao Paulo, SP, Brazil.

**Keywords:** Scaphoid Bone, Pseudarthrosis, Bone Plates, Bone Transplantation, Autografts, Osso Escafoide, Pseudoartrose, Placas Ósseas, Enxerto Ósseo, Autoenxertos

## Abstract

**Objective:**

To make a comparative analysis of patients with scaphoid pseudarthrosis operated with screw and corticocancellous graft and patients operated with plate and cancellous graft only, in regards to consolidation, carpal stability and limb functionality.

**Methods:**

non-randomized retrospective cohort study. Nineteen patients with scaphoid pseudarthrosis without advanced collapse were included in the study, of which 9 patients operated with screw and corticocancellous graft (Group A) and 10 operated with plate using cancellous graft (Group B). The following were evaluated preoperatively and 12 weeks postoperatively: functional recovery using the visual analogue scale, range of motion, grip strength, digital pinch strength, DASH and MAYO wrist score functional scales. To assess carpal instability, the scapholunate and radiolunate angles were assessed on radiographs and the interscaphoid angle on CT. And the bone consolidation rate was assessed with CT in the 8th postoperative week.

**Results:**

group A with 90% and B with 100% consolidation rate, however the latter with a longer average time for consolidation - 9.7 weeks (p = 0.002). Improvement in pain intensity was achieved in both groups (p = 0.03). Increased pinch strength (p=0.04) and grip strength in group B and decreased in group A. The range of motion was superior in group B, with loss of ulnar deviation (p=0.02) and radial deviation (p=0.007) in group A. Regarding the MAYO wrist score, there was loss of function in group A and an increase in group B (p=0.007). There was correction of the scapholunate angle in both groups (p=0.03), with no difference between them.

**Conclusions:**

Patients in group B had better recovery of range of motion, pinch and grip strength, and better functionality according to the MAYO wrist score. ***Level of Evidence III; Study with an Almost-Experimental Design as a Non-Randomized Study with a Single Pre- and Post-Test Group. (Non-Randomized Retrospective Cohort).***

## RESUMO

**Objetivo:**

comparar pacientes com pseudoartrose do escafóide operados com parafuso e enxerto córtico esponjoso e pacientes operados com placa e enxerto somente esponjoso em relação à consolidação, estabilidade carpal e funcionalidade do membro.

**Métodos:**

trabalho de coorte retrospectivo não-randomizado. Foram incluídos no estudo 19 pacientes com pseudoartrose do escafóide sem presença de colapso avançado, sendo 09 pacientes operados com parafuso e enxerto córtico esponjoso (Grupo A) e 10 operados com placa utilizando enxerto esponjoso (Grupo B). Foram avaliados no pré-operatório e com 12 semanas de pós-operatório: recuperação funcional através da escala visual analógica, amplitude de movimento, força de preensão, força de pinça digital, escalas funcionais de DASH e MAYO wrist score. Para avaliar a instabilidade carpal foram avaliados os ângulos escafolunar e radiolunar nas radiografias e o ângulo interescafóide na tomografia computadorizada (TC). E a taxa de consolidação óssea foi avaliada com TC na 8a semana pós-operatória.

**Resultados:**

grupo A com 90% e B com 100% de taxa de consolidação, porém este com maior média de tempo para consolidação- 9,7 semanas (p = 0,002). Melhora da dor em ambos os grupos (p=0,03). Aumento de força de pinça (p=0,04) e preensão no grupo B e diminuição no grupo A. O arco de movimento foi superior no grupo B, com perda de desvio ulnar (p=0,02) e desvio radial (p=0,007) no grupo A. Em relação ao MAYO wrist score, houve perda de função no grupo A e aumento no grupo B (p=0,007). Houve correção do ângulo escafolunar em ambos os grupos (p=0,03), sem diferenças entre si.

**Conclusões:**

pacientes do grupo B obtiveram melhor recuperação de arco de movimento, força de pinça e de preensão e melhor funcionalidade de acordo com o MAYO wrist score. ***Nível de Evidência III; Estudo com Delineamento quase-Experimental como Estudo sem Randomização com Grupo Único pré e Pós-Teste (Coorte Retrospectiva não-Randomizada).***

## INTRODUCTION

Scapular fractures account for 60% of carpal fractures, including a high incidence in the overall rate of fractures that occur in the wrist,^1-3^ being the waist region the most prevalent.^4^

Consolidation rates, when properly treated, reach almost 95%.^5^ However, a neglected or not properly treated scaphoid fracture has non-consolidation rates around 5-10%.

Some factors related to the type of fracture such as: deviation, instability, proximal pole; can raise this percentage of non-union to 90%.^5^

The pseudarthrosis of the scapula represents a challenge to traumatic handles. The evolution to advanced carpal collapse (SNAC) leads to a significant decrease in wrist function as well as quality of life, as this evolution is associated with constant pain.

In terms of anatomy and biomechanics, the scaphoid tends to flex in relation to the semilunar, while the pyramid tends to extend. After scaphoid pseudarthrosis, the proximal pole tends towards extension, since it is connected to the proximal carpal row by the scapholunate (SL) and dorsal inter metacarpal (DIC) ligaments, while the distal pole tends to adopt a flexion deviation. This biomechanical imbalance leads to humpback deformity of the scaphoid and DISI deformity (Dorsal Intercalated Segment Instability).^6^

The objectives of the treatment of of the scaphoid are: to correct the normal alignment of the scaphoid to restore the biomechanical of the wrist and to obtain rigid stabilization of the fragments with osteosynthesis, using vascularized or non-vascularized graft.^6-8^ If the general principles of treatment are not applied, the kinematic alteration of carpal bones can lead to arthrosis, producing pain and decreased function.^9-12^

Regarding the biomechanical aspects in the comparison between plate and screw, there is no difference between the two methods when used in bones with standard densities, however, in cases of low bone density, the plate proved superior.^13^ In a study conducted on corpses, the plate also proved more effective, showing greater rigidity when load applied.^14^

In general terms, the use of the plate for the treatment of pseudarthrosis of the scaphoid has some advantages: greater rotational stability and rigidity,^15^ gold standard if reoperation is needed,^16,17^ and for cases with instability,^18^ effective humpback correction,^19^ favorable postoperative DASH score,^17^ and is effective if there is focal cominution.^20^

Some disadvantages of using the plate include: longer period of post-operative immobilization due to the risk of impact of the plate with the flying surface of the radio,^21^ not suitable for pseudarthrosis of the proximal pole due to the risk of impact,^19^ lower amplitude of movement and grip force in the first 3 months of post-operative.^22^

In screw fixation, the tri cortical cancellous cortical graft plays a fundamental role in the correction of humpback deformity, in the maintenance of the anterior support and in the alignment of the scaphoid as described by Fernandez et. al. (1990).^23^

There is still no consensus as to whether the new method of plaque fixation using only cancellous graft is superior to the conventional method described by Fernandez et. al. (1990).^23^

This study aims to compare patients with pseudarthrosis of the scaphoid operated with screw using cancellous cortical graft with patients operated with plaque using cancellous graft only regarding:

- Consolidation time;
- Humpback correction;
- Pain, amplitude of movement, grip force, pinch strength and functional scales;
- Complications: Infection, plate impact, stiffness.

## MATERIALS AND METHODS

This study was conducted at the IOT - HC/FMUSP. Non-randomized retrospective cohort work. The study included 10 patients operated with screw and cancellous cortical graft (Group A) and compared with 10 patients operated with plaque, however, using only cancellous graft (Group B). The patients were operated between January 2018 and May 2023, by different surgeons, all seniors and familiar with the surgical technique.

Inclusion criteria:

- Pseudoarthrosis of Alnot^24^ type IIB and IIIA squamous collar (Table 1)
- No prior surgery
- No other injury to the upper limb
- Minimum of 6 months of development

**Table 1:** Classification of pseudarthrosis according to the Alnot system.^25^

Grade I		No linear union, no alteration of the shape of the scaphoid, instability or poor carpal alignment
Grade II	II A	No stable bond with small bone reabsorption on the fracture line, no instability or poor carpal alignment.
	II B	Non-mobile bond with anterior defect and proximal pole flexion under the tubercle of the scaphoid, with the presence of DISI
Grade III	IIIA	Non-mobile bond with carpal instability or poorly aligned redutile with radial-styloid arthrosis isolated
	III B	Non-mobile bond with deviation and instability or poorly aligned redutile, with fossa arthrosis of the scaphoid or intracarpal
Grade IV	IV A	Necrosis of the proximal fragment with poor carpal alignment
	IV B	Necrosis of proximal fragment with fossa arthrosis of the scaphoid or intra carpal

Exclusion criteria:

- Loss of patient tracking.
- Advanced degenerative framework - SNAC type III
- Cases without all imaging tests
- Cases without functional evaluations in the scheduled time

The pre- and postoperative clinical evaluation data were evaluated for:

- Pain intensity (analog visual scale)
- Amplitude of movement (goniometry)
- Stretch force (Jamar)
- Digital pinch strength
- Functional scales of DASH and MAYO wrist score,^26^ in preoperative and 12 weeks postoperative

They were also evaluated radiographically in pre and postoperative with:

- X-rays of both wrists in the incidences: front, with ulnar deviation, profile and obliques in the preoperative, with 3, 6 and 12 weeks postoperative; where the escapular and radiolunar angles were measured.
- Preoperative computed tomography (TC) to evaluate the presence of carpal collapse and measure the inter scaphoid angle
- CT with 8 weeks of postoperative to evaluate consolidation.

Presence of bone bridge between the graft and the proximal and distal poles at 8 weeks was considered a consolidation criterion in the evaluation. In the absence of this finding, the patient was re-evaluated every 4 weeks until showing signs of consolidation in CT.

In the postoperative period, it was established the use of gessed tail or antebrachiopolegar orthesis until the presence of consolidation as a rehabilitation protocol.

### Surgical technique

**Figure 1:**
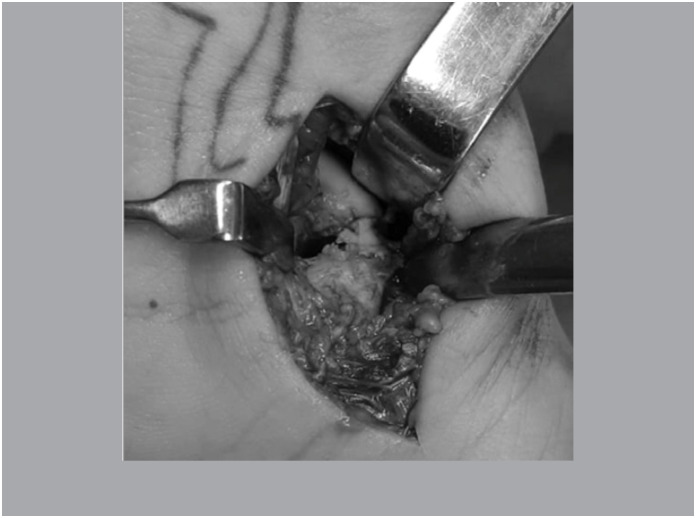
Volar view showing the focus of pseudarthrosis.

Source: Photo by Erick Yoshio Wataya, Institute of Orthopaedia and Traumatology of HCFMSP, São Paulo, Brazil.

Patients in horizontal dorsal decubitus undergoing general anesthesia and regional blockage, inflated pneumatic tourniquet at 250 mmHg. Via volar in the wrist (via de Russe), with capsulotomy for access to the joint and the scapula, preserving ligament insertion. (Figure 1)

**Figure 2:**
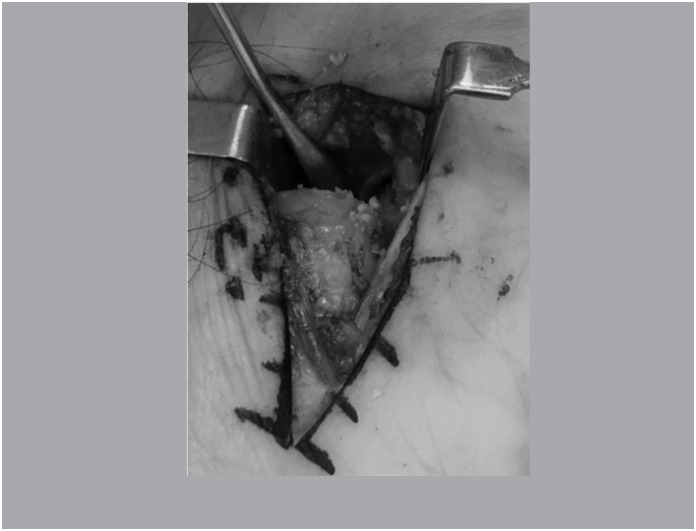
Resecated pseudarthrosis focus demonstrating viable bone.

Source: Photo by Erick Yoshio Wataya, Institute of Orthopaedia and Traumatology of HCFMSP, São Paulo, Brazil.

With the scaphoid exposed, the focus of pseudarthrosis was cruentized until viable bone with good vascularization was present. (Figure 2)

**Figure 3:**
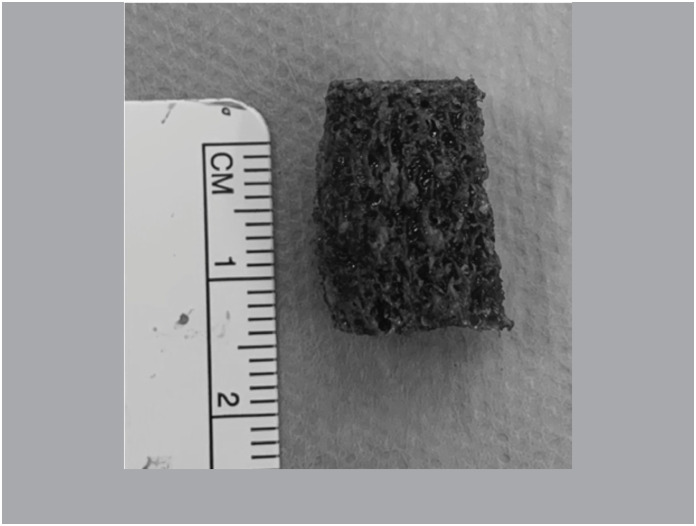
Structured only cancellous bone graft after cortical bone resection.

Source: Photo by Erick Yoshio Wataya, Institute of Orthopaedia and Traumatology of HCFMSP, São Paulo, Brazil.

Performed reduction of the scaphoid and calculated the size of the graft required. Structured cancellous cortical bone graft from the iliac (Group A) was extracted. In Group B the tri cortical area was removed, remaining only the cancellous graft in a block, in a structured way. (Figure 3)

**Figure 4:**
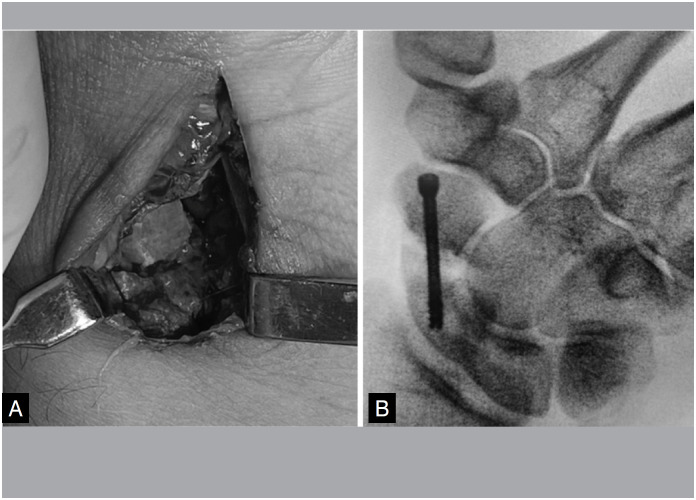
A) Cancellous cortical graft in the focus of pseudarthrosis (Group A). B) Fixation with canulated screw.

Source: Photo by Erick Yoshio Wataya, Institute of Orthopaedia and Traumatology of HCFMSP, São Paulo, Brazil.

The cancellous cortical graft was inserted into the bone defect and fixed with 2.2mm Speed Tip Cannulated Compression Screws (CCS) with radioscopy. (Figure 4A and 4B)

**Figure 5:**
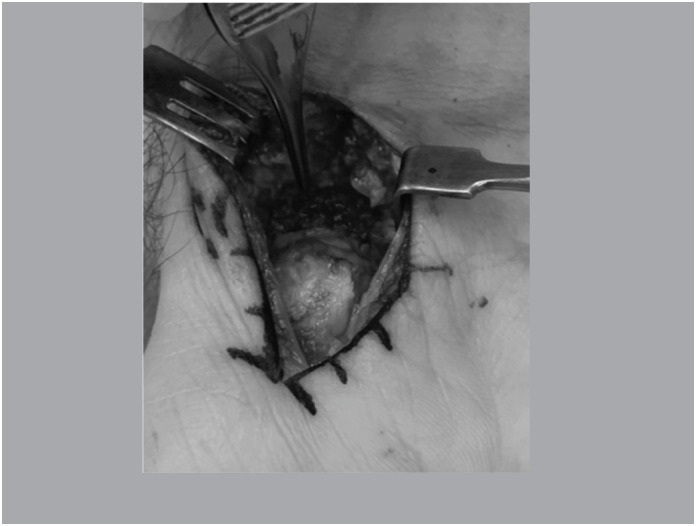
Cancellous-only graft in the focus of pseudarthrosis (Group B).

Source: Photo by Erick Yoshio Wataya, Institute of Orthopaedia and Traumatology of HCFMSP, São Paulo, Brazil.

**Figure 6:**
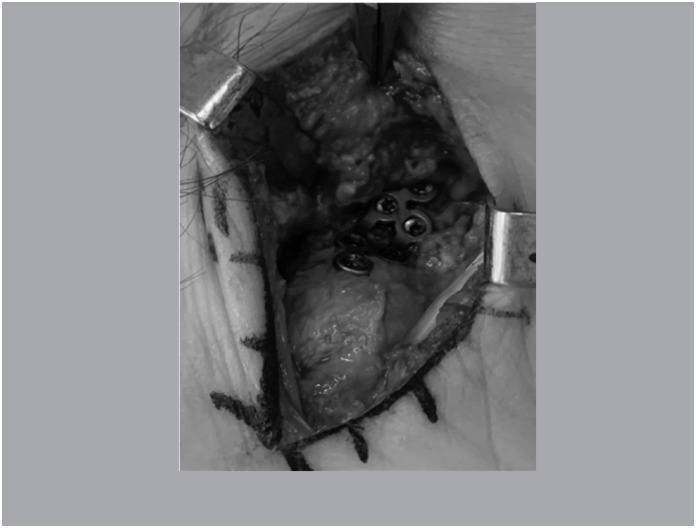
Osteosynthesis of the escafoid with blocked plate.

Source: Photo by Erick Yoshio Wataya, Institute of Orthopaedia and Traumatology of HCFMSP, São Paulo, Brazil.

In group B, only cancellous graft was used in the focus of pseudarthrosis (Figure 5) and the fixation with low profile Tri-lock"® plates of 1.5 mm for Medartis scaphoid. (Figure 6)

After the osteosynthesis, joint mobility test and capsuloraphia were performed on the synthesis. Disinflated the tourniquet, carried out hemostasis, cleaning and suturing by planes.

### Statistical analysis

Based on the study by Beaton et al. (2001),^27^ was defined the clinically important minimum difference (MCID – Minimum clinically important difference) for one of the following variables: obtaining consolidation, correction of deformity of the scapula and MAYO wrist score. A 5% alpha error and 80% statistical power were established. There are no prospective works of this nature, which makes the sample calculation difficult. We chose a convenience sample of 20 patients.

The quantitative data were submitted to the evaluation of normal distribution by the Shapiro-Wilk test and expressed as average, standard deviation, median, maximum and minimum values and sample size. For the comparison between quantitative data, the Student T test was used when normal (parametric) distribution and the Mann-Whitney U test for nonparametric data.

The qualitative data were demonstrated as frequencies and percentiles. For the comparison between qualitative data, we will use the qui-square test.

In the data comparison, a significance level of 5% (alpha = 0.05) was used, and the values of p < 0.05 were considered statistically significant.

### Ethical Approval

This study was approved by the Institutional Ethics Committee under the opinion number 6.877.718 and the Terms of Free and Informed Consent was signed by all participants prior to the study.

## RESULTS

19 patients were included in the study, including 09 from Group A (glass with cancellous cortical graft) and 10 from Group B (plaque with cancellous graft). One patient in Group A was excluded for loss of follow-up. The mean time from the date of the trauma to the date of surgery was 14 months for group A and 19 months for group B. 05 patients in Group A had pseudoarthrosis of the escafoid in their dominant hand, which occurred in 07 of the 10 patients in Group B. (Tables 2 and 3)

**Table 2:** Epidemiological profile of the group of patients fixed with plaque + cancellous graft.

Patient	Dominance	Side Operated	Profession	Time since the trauma	Trauma mechanism
1	Right-handed	Right	Driver	8 months	Entorse of hand
2	Right-handed	Left	Manager of confectionery	6 months	Fall
3	Right-handed	Right	Mason	1 year and 6 months	Height drop (roof)
4	Right-handed	Left	Seller	2 years	Falling during sports (football)
5	Right-handed	Left	Machine Operator	1 year and 2 months	Motorcycle fall
6	Right-handed	Right	Glassman	1 year	Fall
7	Right-handed	Right	Delivery	2 years	Motorcycle fall
8	Right-handed	Right	Shelf storage	6 months	Falling during sports (basket)
9	Right-handed	Left	Unemployed	1 year and 5 months	Motorcycle fall
10	Right-handed	Left	Secretary	1 year and 8 months	Falling during sports (football)

**Table 3:** Epidemiological profile of the group of patients fixed with screw + cancellous cortical graft.

Patient	Dominance	Side Operated	Profession	Time since the trauma	Trauma mechanism
1	Right-handed	Right	Delivery	6 years	Motorcycle Accident
2	Right-handed	Right	Student	6 months	Motorcycle Accident
3	Right-handed	Left	Machine Operator	2 years	Fall
4	Right-handed	Right	Entrepreneur	1 year and 8 months	Motorcycle Accident
5	Right-handed	Right	Manual worker	1 year and 9 months	Fall
6	Right-handed	Left	Police	9 months	Falling during sports (football)
7	Right-handed	Left	Seller	1 year and 8 months	Fall
8	Left-handed	Left	Security	8 months	Fall
9	Right-handed	Right	Unemployed	1 year and 9 months	Motorcycle Accident
10	Right-handed	Right	Manual worker	1 year and 1 month	Fall

**Figure 7:**
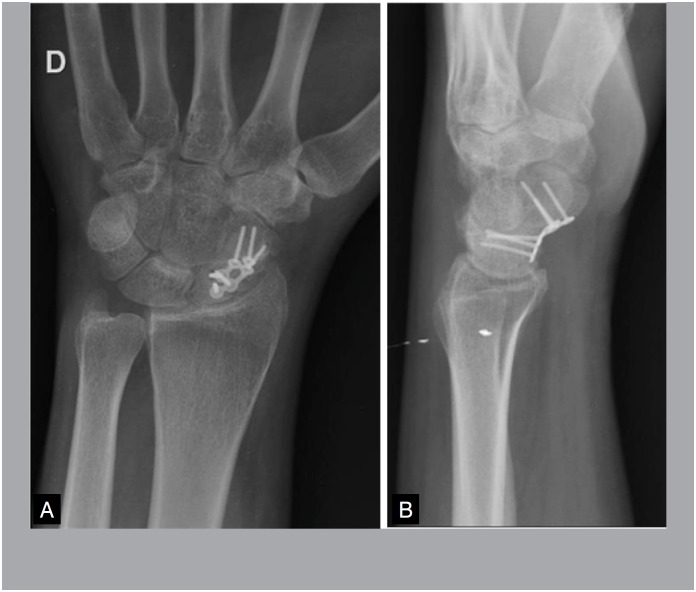
Postoperative X-ray of pseudoarthrosis fixation.

Source: Photo by Erick Yoshio Wataya, Institute of Orthopaedia and Traumatology of HCFMSP, São Paulo, Brazil.

**Figure 8:**
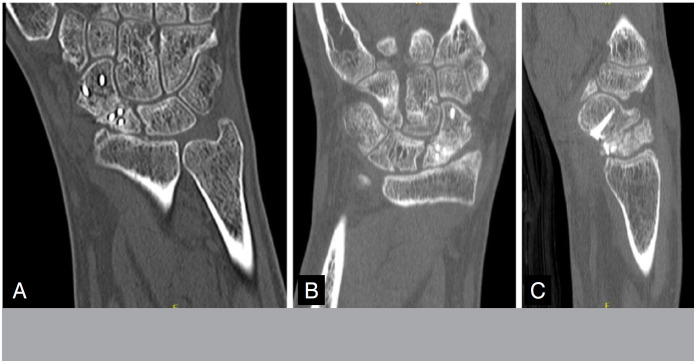
Postoperative pseudoarthrosis fixation tomography with 02 months of evolution, still without signs of bone bridge. B and C: postoperative tomography of pseudoarthrosis fixation with 03 months of evolution, with presence of bone bridge in the coronal plane and flight in the sagital plane.

Source: Photo by Erick Yoshio Wataya, Institute of Orthopaedia and Traumatology of HCFMSP, São Paulo, Brazil.

There was one case of non-consolidation in Group A due to implant failure. In the other cases, pseudoarthrosis was consolidated in CT at 08 weeks. All cases in Group B consolidated, however, in two cases, there was only evidence of consolidation in CT at 12 weeks (Figure 7 and 8) and one case at 16 weeks, with an average of 9.7 weeks, with statistical significance (p = 0.002).

In relation to the dynamometry, the grip strength of the wrist, the digital and triple pinch strength were evaluated. In the preoperative evaluation, the initial measurements of strength were higher in Group A. Thus, the comparison was chosen in relative and non-absolute values. At the end of the evaluation, the average grip and pince strength were higher in Group B (p=0.04). There was loss of grip and pinch strength in Group A in the postoperative evaluation, even after the consolidation of pseudarthrosis. Already in Group B, there was gain in all dynamometry parameters.

On average, there was a loss of 14% of grip strength in patients in Group A and a gain of 27% of grip strength in patients in Group B in the post-operative evaluation. Regarding pinch strength, there was a loss of 12% for Group A and a gain of 31% for Group B. There was a gain of 47% triple pinch for both groups.

The goniometry was evaluated in four parameters: flexion, extension, ulnar deviation and radial deviation of the wrist, and the relative values were considered for comparison. Even after the consolidation of pseudoarthrosis, there was loss of movement arc in all criteria of Group A and gain in all criteria of Group B. In relation to Group A, there was loss of 30% of wrist extension, 16% of flexion, 14% of ulnar deviation (p=0.02) and 13% of radial deviation (p=0.007). In relation to Group B, there was an increase of 29% in the extension of the wrist, 18% in the flexion, 21% in the ulnar deviation and 75% in the radial deviation.

**Figure 9:**
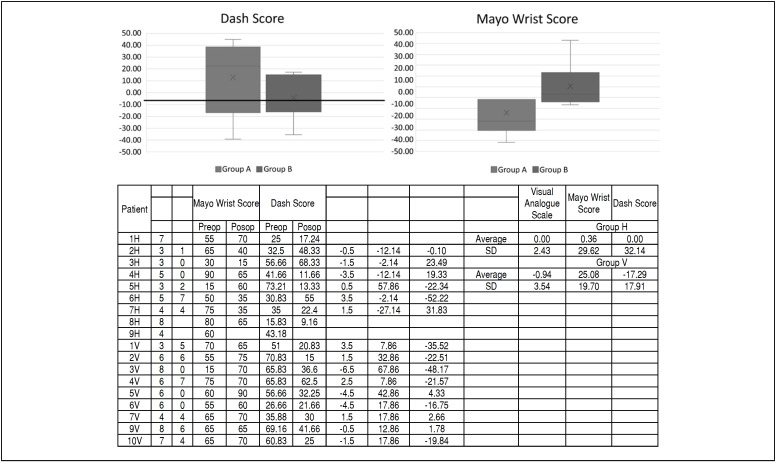
Comparison between groups by DASH and Mayo wrist score after 12 weeks.

Patients were evaluated by MAYO wrist score in preoperative and 12 weeks postoperative. There was a 14% deterioration in Group A score and a 52% improvement in Group B score (p=0.007). In relation to the DASH score, there was improvement in the parameters in both groups, being 14% in group A and 40% in group B. (Figure 9)

There was an improvement in the intensity of pain in both groups in the Analogous Visual Scale evaluation (p=0.03), with no significant difference between the groups.

Pre- and post-operative radiographic parameters were evaluated, with emphasis on the scapular angle due to its importance for the correct reduction of the scapula and consideration for carpal stability. There were two cases in each group where the angle, even in the postoperative X-rays, was above the normal range. In relation to the overall average, in Group A there was reduction of the angle from 71° to 60° (upper limit of normality), with statistical significance (p=0,03), and in Group B reduction from 57° to 47°. In relation to the radiolunar angle, used to evaluate the correction of deviation in DISI, there was improvement in parameters in both groups, with no significant difference. In Group A, the preoperative average angle was 17.6°, with correction to 13.75° in the postoperative period. Group B showed improvement from 22° to 19° on average in the postoperative period.

Another parameter for evaluating postoperative reduction is the interescafoid angle, measured in the sagital plane of preoperative CT and with 08 weeks of evolution. There was a significant improvement in the angle in all cases operated, with one case of Group B keeping values above the normal consideration, but with expressive improvement. In Group A, the average angle values were 52° in preoperative and 17° in postoperative. In Group B, the reduction was from 50° to 34° after surgery.

## DISCUSSION

In relation to the post-operative evolution in the first 12 weeks, both for movement arc and for the evaluation of strength and function of the hand, a superiority of the plate compared to the use of the screw was observed, with better rates in functional evaluations and early return to activities. It is possible that the anterior support in the escafoid provided by the plate and the improvement in angular stability in the correction of carpal alignment angles have provided a clinical improvement in strength and mobility parameters.

The volar plates for pseudarthrosis of scaphoid were described in 1993 by Braun et al.^28^ There are studies that evaluated the use of scaphoid plates in complex cases of pseudarthrosis after fixation with auto-compressive screws and cases with severe deformity of *humpback.* ^16,18,29,30^ Esteban-feliu et al described cases with use of volar plates with 87% consolidation rate in 15 patients.^25^

Regarding the consolidation rates in the cases of osteosynthesis with screws and cancellous cortical graft, the conventional method for primary pseudarthrosis, the literature demonstrates good consolidation rates, around 84 to 95%,^31^ similar to our study, in which 90% of cases consolidated.

Ghoneim described 14 cases of plaque-treated pseudarthrosis, with an average of 16.5 months of evolution, with a 93% consolidation rate, which took an average of 3.8 months.^18^ Other studies show rates ranging from 72%,^32^ to 100%,^16^ with plaque use. In our study, we obtained a 100% consolidation rate, similar to the data demonstrated.

When compared, the two methods show no statistical difference in consolidation rate,^32^ thus being equally effective in bone consolidation, however, the cases fixed with plaque and cancellous graft showed faster consolidation and greater correction of the *humpback.* ^33^

The pure cancellous graft, due to its osteogenic, osteoinductive and osteocondutory capacity,^34^ showed superior consolidation rates when used with screw osteosynthesis.^35^ In the cases associated with plaque osteosynthesis, in addition to the satisfactory consolidation rates, it also presented better angular correction of the scaphoid and better functional return of pinch strength and grip.^36^ Putnam et al^37^ presented the best consolidation rates in cases of pseudarthrosis of the scaphoid, using plaque fly with cancellous graft, with 100% consolidation rate in 26 cases, with up to 18 weeks of follow-up.

The anatomical shape of the squamous plate has been shown to be useful for the reduction of pseudarthrosis fragments, especially in the correction of the *humpback* and other deformities, in addition to allowing better support for the graft when it is not cortical. Another concern with the use of self-compressive screws is the possible rotational deviation of the escape, which can be avoided with the plate.

**Figure 10:**
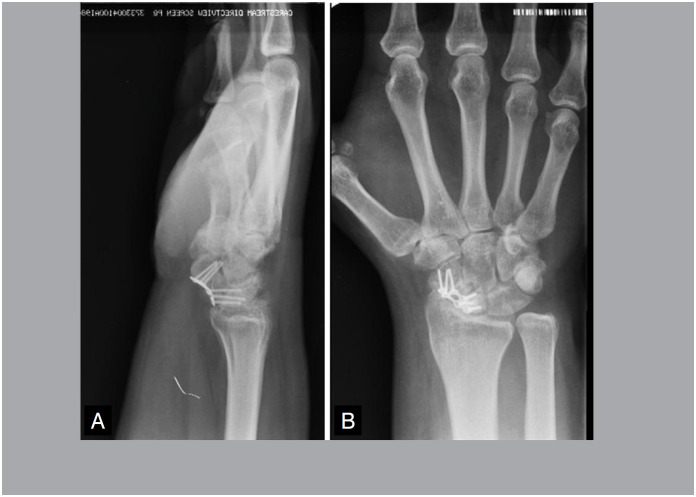
Postoperative pseudarthrosis fixation X-ray of the scapula.

Source: Photo by Erick Yoshio Wataya, Institute of Orthopaedia and Traumatology of HCFMSP, São Paulo, Brazil.

**Figure 11:**
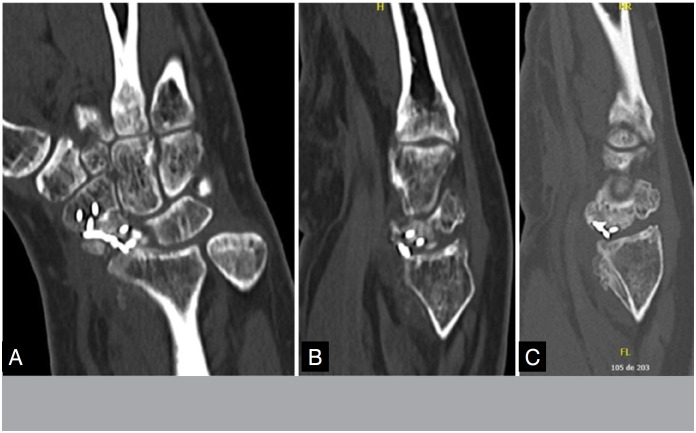
Postoperative pseudarthrosis arthrosis fixation tomography of the proximal pole of the scaphoid with 08 weeks of evolution, evidencing the impact of the plaque on the volar surface of the radio.

Source: Photo by Erick Yoshio Wataya, Institute of Orthopaedia and Traumatology of HCFMSP, São Paulo, Brazil.

A disadvantage described with the use of the board is the friction of the board with the flying surface of the radio. Although the low profile of the plaque mitigates this complication, the clinical experience of the study showed that if it is not properly positioned on the scapula, it can cause friction on the flying edge of the radio, especially to grip and flexion of the wrist. In cases of non-consolidation of the proximal pole of the escafoid, friction of the plate with the radio may occur in an attempt to fit the plate for fixation of the proximal pole fragment. In one case of group B, although the fracture was from the neck of the scapula, for better fixation and reduction, the plate was positioned in a more proximal topography, causing friction with the radio (Figure 10 and 11). In this case, the patient evolved with pain to the flexion of the wrist and the plaque was removed after consolidation.

**Figure 12:**
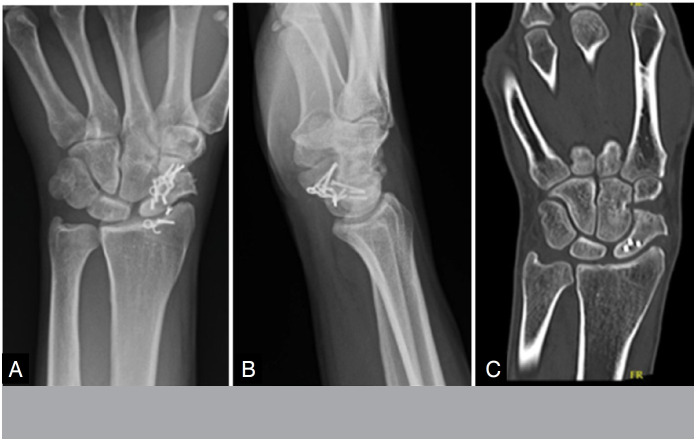
X-rays showing plaque C fracture: tomography with evidence of bone consolidation in the case of plaque fracture.

Source: Photo by Erick Yoshio Wataya, Institute of Orthopaedia and Traumatology of HCFMSP, São Paulo, Brazil.

There were 02 cases of plaque-related complications: 01 case of plate rupture and 01 case of plate screw release, both after pseudarthrosis consolidation (Figure 12). Esteban-feliu et al. reported 04 cases of complications associated with the plaque, including 01 cases of plaque breakage and 03 cases of release of screws from the plaque. This study followed patients for 03 years.^30^

Other symptoms described when using the plate are pain to mobilize the wrist, stinging and cold intolerance.^19^ Although there were no complaints of stinging, post-operative pain was a common complaint, although there was no statistical difference between the mean pain, taking into account the Analogous Visual Scale (p=0.03) in both groups.

The study presented limitations in relation to the short follow-up time of the patients, which may have been insufficient to define late complications, especially in relation to the use of the plate with the impact and pain when flirting the wrist. The sample was also small and insufficient to determine comparisons with statistical significance in some of the parameters used. The surgeries were performed by different surgeons, all senior and familiar with the technique, but this can also be considered a limitation of this study.

However, despite the limited sample, this study was able to evaluate parameters that attest to the functionality of the limb after surgery and compare the two groups with statistical significance favorable to the use of the plaque. In addition, it is a study that compares, in an unprecedented way, clinical, functional and radiological aspects of the standard and most common technique through the use of cancellous cortical graft plus fixation with screw; with the latest technique, using cancellous graft plate.

## CONCLUSION

The present study favors the use of cancellous graft plates over the screw and cancellous cortical graft technique with consolidation rates in 100% of cases, but with a higher average time for consolidation. Patients using the plate obtained better postoperative recovery, with greater movement arc, better pinch recovery and grip strength and better functionality according to MAYO wrist score.
